# survivalContour: visualizing predicted survival via colored contour plots

**DOI:** 10.1093/bioadv/vbae105

**Published:** 2024-07-25

**Authors:** Yushu Shi, Liangliang Zhang, Kim-Anh Do, Robert R Jenq, Christine B Peterson

**Affiliations:** Department of Population Health Sciences, Weill Cornell Medicine, New York, NY 10065, United States; Department of Population and Quantitative Health Sciences, Case Western Reserve University, Cleveland, OH 44106, United States; Department of Biostatistic, The University of Texas MD Anderson Cancer Center, Houston, TX 77030, United States; Department of Genomic Medicine, The University of Texas MD Anderson Cancer Center, Houston, TX 77054, United States; Department of Biostatistic, The University of Texas MD Anderson Cancer Center, Houston, TX 77030, United States

## Abstract

**Summary:**

Advances in survival analysis have facilitated unprecedented flexibility in data modeling, yet there remains a lack of tools for illustrating the influence of continuous covariates on predicted survival outcomes. We propose the utilization of a colored contour plot to depict the predicted survival probabilities over time. Our approach is capable of supporting conventional models, including the Cox and Fine–Gray models. However, its capability shines when coupled with cutting-edge machine learning models such as random survival forests and deep neural networks.

**Availability and implementation:**

We provide a Shiny app at https://biostatistics.mdanderson.org/shinyapps/survivalContour/ and an R package available at https://github.com/YushuShi/survivalContour as implementations of this tool.

## 1 Introduction

Over the past few decades, there have been major advances in survival modeling, including the development of approaches for handling competing risks and machine learning methods for survival prediction. However, there is a lack of visualization tools for translating these cutting-edge models into effective graphical representations. Even for classical models, such as the Cox or Fine–Gray methods, there is a gap between the model outputs and the presentation of results.

**Figure 1.**
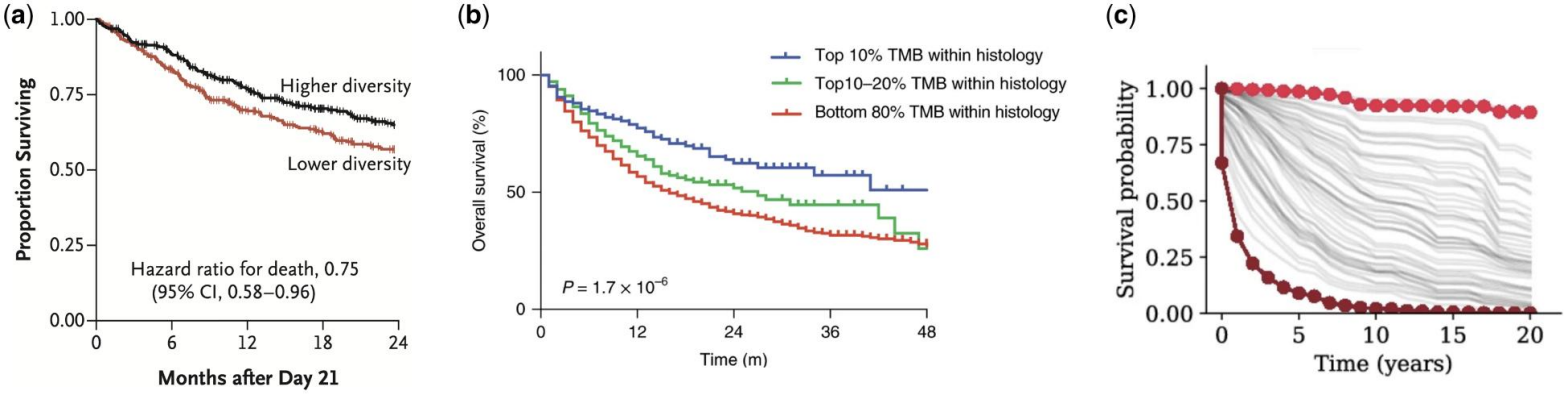
Existing approaches for survival model visualization depicting two groups (a), multiple groups (b), or individual survival curves (c).

A typical visualization approach in current practice is to split the covariate of interest at its median and present Kaplan–Meier curves for subjects with low versus high values. This approach has significant shortcomings, as precise information from the continuous predictor gets lost during binarization (Altman and Royston 2006). In addition, it creates a divergence between the analysis and the visual presentation. This inconsistency is clear in Fig. 1a, which originally appeared in Peled *et al.* (2020). This study established that lower microbiome diversity was associated with increased risk of mortality following stem cell transplant. In the presentation of the data shown in Fig. 1a, patients were separated into low versus high diversity groups based on the median value of diversity for samples collected between Days 7 and 21. The Kaplan–Meier curves for each group were presented alongside the hazard ratio for death from a Cox model with log10(diversity) as the primary predictor of interest.

To highlight trends for extremes of the predictor distribution, narrower categories may be constructed using quantiles of the predictor. However, this step introduces an additional element of subjective data exploration in choosing how to represent the association between the continuous predictor and survival. Figure 1b depicts an example where tail quantiles were identified to highlight differences in the survival curves. In this figure, which originally appeared in Samstein *et al.* (2019), the continuous predictor is tumor mutational burden (TMB), which has been associated with clinical benefit from immune checkpoint inhibitors. The authors note that a variety of cutpoints could be chosen to define the high TMB group; in Fig. 1b, they illustrate survival curves for patients in the top 10%, top 10%–20%, and bottom 80% of TMB values within each cancer type.

The lack of informative visualization approaches becomes even more prominent when considering machine learning methods for survival prediction, such as the random survival forest (Ishwaran *et al.* 2008). Recently, there has been a flurry of proposals on the use of deep learning for this purpose, including the DeepHit (Lee *et al.* 2018) and DeepSurv (Katzman *et al.* 2018) methods. The challenge of presenting survival predictions generated by these models is particularly pressing given the complex nonlinear relationships they encode.

Spaghetti plots are one existing approach to visualize survival model outputs. In a spaghetti plot, a distinct trajectory, or “noodle,” can be used to display predicted survival for each subject. Figure 1c highlights the use of a spaghetti plot to display predicted survival from a deep learning model for cancer survival prediction, which originally appeared in Vale-Silva and Rohr (2021). In this figure, each curve corresponds to the predicted survival for a specific kidney cancer patient; the bold curves represent outlier patients with the most extreme survival predictions. Although the curve for an individual subject may be informative, when applied to a patient cohort, spaghetti plots tend to result in a tangle of curves without shedding light on the link between covariate values and survival.

Alternative approaches to visualization have been proposed. In particular, the idea of visualizing survival outcomes through a contour plot appeared in Lumley and Heagerty (2000). However, their method was implemented in XLisp-Stat, which is not widely used today, and does not reflect developments in the field of survival analysis over the past two decades. Currently available tools for survival visualization include the survminer package (Kassambara *et al.* 2021), which can produce publication-ready plots, but still focuses on the display of Kaplan–Meier and cumulative incidence curves. Most recently, a survival area plot for depicting predictor effects within the causal inference framework has been proposed (Denz and Timmesfeld 2023). However, there remains a lack of user-friendly tools for presenting the results of popular survival models.

## 2 Methods

To address this gap, we have designed a Shiny app and R package for interactive visualization of survival predictions. Both tools enable the production of visually effective plots and offer flexible options for competing risks and interval censoring, while the R package captures more advanced models for survival association including deep learning models.

### 2.1 Shiny app

**Figure 2.**
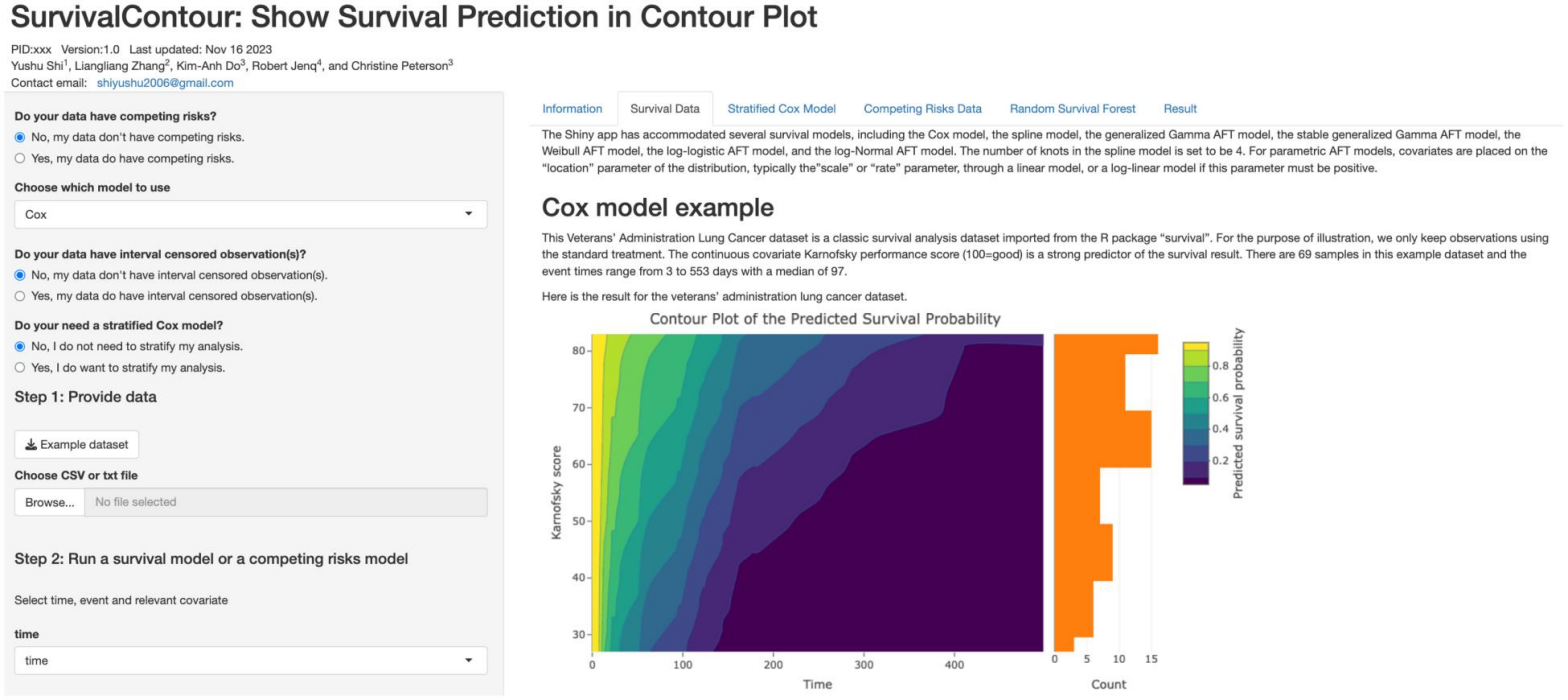
A snapshot of the survivalContour Shiny app.

**Figure 3.**
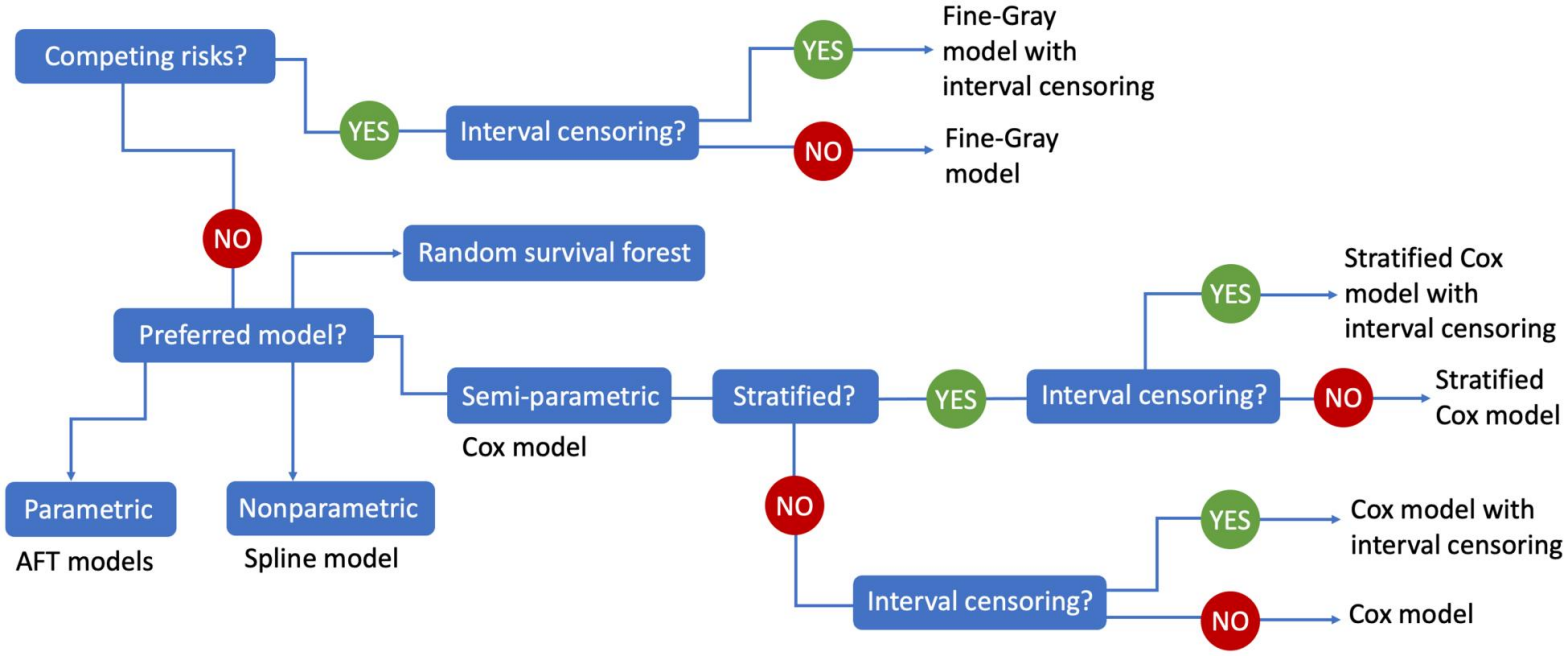
Decision tree implemented in the Shiny app.

The survivalContour Shiny app is available online at https://biostatistics.mdanderson.org/shinyapps/survivalContour/. In the Shiny app, we guide users in selecting an appropriate survival model for their data. A snapshot of the app is shown in Fig. 2. There are options for settings with competing risks, where the event of interest may be precluded by a different event, and for interval-censored data, where the event time is only known to occur within a given window. Competing risks and interval censoring are both common in biomedical studies. For example, competing risks occur when a patient is no longer at risk for cancer progression because they have died due to an infection. Interval censoring occurs whenever there are fixed time points for screening or follow-up. For example, in a cancer screening study, if a scan reveals that the patient developed a tumor since their last visit, the true event time lies somewhere during the intervening time window but is not exactly observed. For settings without competing risks, we offer parametric, nonparametric, or semiparametric survival models, as these may be preferred for different applications. Parametric models, which assume a known distribution for the survival times, offer an advantage for settings where out-of-sample prediction is a priority (Jackson 2016). Nonparametric survival models, in particular spline-based methods, offer greater flexibility on the shape of the hazard (Royston and Parmar 2002). Finally, semiparametric methods, in particular the Cox model, which avoids assumptions on the baseline hazard function, are a mainstay in applied survival analysis. We offer both the classic and stratified Cox model, which allows the baseline hazard function to differ across levels of a covariate. These options are illustrated as a decision tree in Fig. 3. More complex models, in particular methods based on deep neural networks, require the specification of a larger number of parameters and careful fine-tuning; for this reason, we defer these options to the R package.

Once the desired model has been selected, we ask users to upload their data, designate the time and censoring indicator, and select the continuous predictor for visualization. We then produce a 2D contour plot: in this plot, the *x*-axis is time, the *y*-axis is the continuous predictor of interest, and the intensity of regions in the plot corresponds to the predicted survival probability from the fitted model. A histogram showing the distribution of the continuous predictor is displayed alongside the contour plot. In addition to the major continuous covariate of interest, users can provide other covariates to be included as adjusters in the model. The survival predictions presented in the contour plots are conditional on the covariate values: these may be specified to a desired level by the user, or taken as the default of the median value for continuous covariates and the most frequent value for categorical covariates.

### 2.2 R package

In addition to the Shiny app, we provide an R package for more advanced users, which allows the display of predictions from deep neural network-based models. The survivalContour R package can be downloaded from https://github.com/YushuShi/survivalContour. We list the models that are compatible with our tools in Supplementary Table S1.

### 2.3 3D plots

Both the Shiny app and the R package support visualization of the survival contours in three dimensions. This provides a more comprehensive understanding of predicted survival, since the 2D colored plot essentially represents a top-down view of the 3D structure. Additionally, the 3D representation allows for the illustration of confidence intervals as semitransparent layers.

## 3 Results

In this section, we illustrate the capacity of our Shiny app and R package to present predictions from the Cox and DeepSurv models. We provide additional applications with the stratified Cox model, the Fine–Gray model, and random survival forests in the Supplementary Material.

### 3.1 Cox model

**Figure 4.**
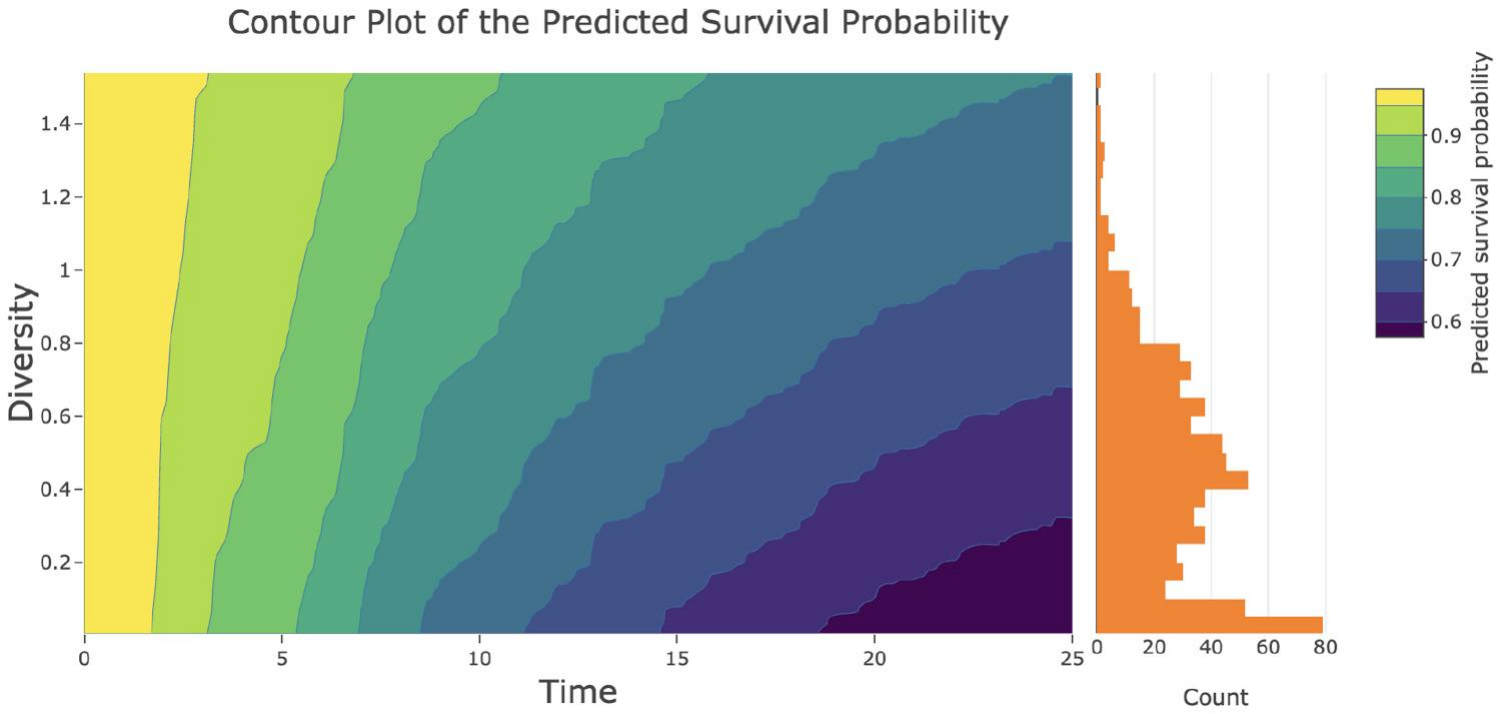
Contour plot created using the survivalContour Shiny app. Diversity corresponds to microbiome diversity on the log10 scale.

To highlight the utility of the Shiny app for presenting the results from a Cox model, we reanalyzed the data presented in Fig. 1a. A richer representation of this association is presented in Fig. 4, which depicts a contour plot of the predicted survival from the Cox model. In this plot, the *x*-axis represents time in months after Day 21, the *y*-axis represents microbiome diversity on the log10 scale, and the color intensity represents the predicted survival probability. As an example of how to interpret the plot, at 10 months, a subject with microbiome diversity < 0.2 on the log10 scale would have a predicted survival probability in the range 70%–75%, while a subject with a diversity value of 1.2 on the log10 scale would have a predicted survival of 80%–85%. This plot corresponds more closely to the analysis performed than the Kaplan–Meier curves and offers an intuitive representation of the strength of association between the predictor and the outcome. The orange histogram at right provides information on the distribution of the predictor values as well as the number of subjects included in the analysis (via the bar heights). Here, the bar heights provide a key insight that very low diversity values (which confer worse expected survival) are in fact quite common in this cohort.

### 3.2 DeepSurv predictions

For this case study, we rely on data collected by the SUPPORT III study (Knaus *et al.* 1995), which aimed to characterize survival outcomes of seriously ill hospitalized adults. This dataset, which contains 8873 observations, was also analyzed in the paper introducing the DeepSurv method (Katzman *et al.* 2018). In this case study, we are interested in depicting the influence of respiratory rate on survival. Additional covariates in the dataset include age, sex, race, the number of comorbidities, the presence of cancer, diabetes, and dementia, mean arterial blood pressure, temperature, white blood count, heart rate, and serum creatine.

**Figure 5.**
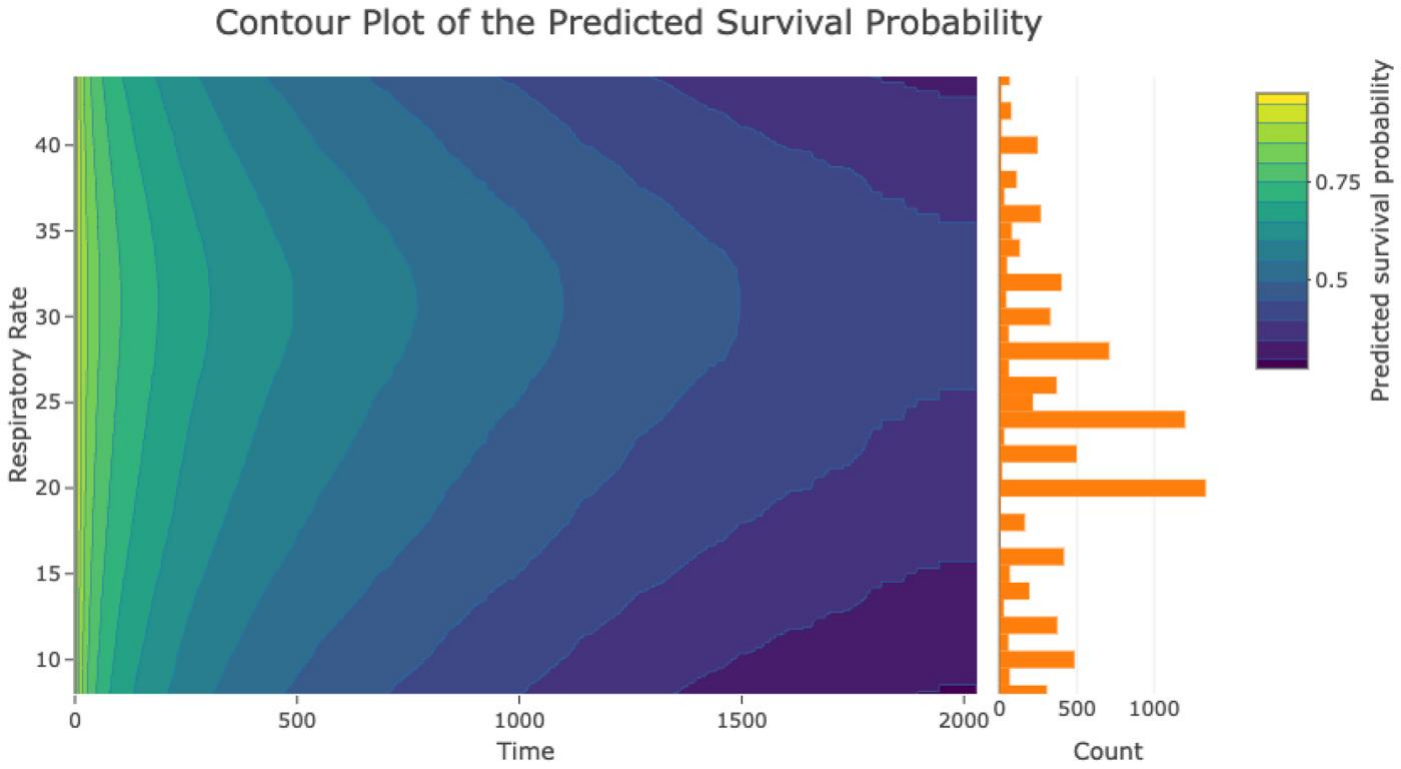
Contour plot created using the survivalContour R package to depict predictions from a DeepSurv model.

Figure 5 depicts a 2D survival contour plot showing the relationship between respiratory rate and predicted survival. A histogram of respiratory rate values is shown to the right of the contour plot. The color variation in the contour plot corresponds to different survival probabilities, with darker colors representing worse predicted survival. What static Fig. 5 cannot reveal is that users can interactively obtain predicted survival rates at specific time points and covariate values by gliding the mouse across the plot. We present the 3D version of this plot in Supplementary Fig. S4. This is also an interactive graphic, which allows users to rotate the 3D surface to view it from multiple angles. Intriguingly, the relation between respiratory rate and survival is not monotone: survival rates deteriorate at extreme respiratory rates, whereas the median respiratory rate forecasts better survival, aligning with clinical intuition. This delicate relationship may not be captured by a basic Cox model, which imposes a multiplicative hazards assumption. Moreover, the common practice of dichotomizing the predictor would fail to identify this relationship. Often, researchers may not be aware of this non-monotone relationship initially. Even if they recognize it and plan to categorize the covariate, determining where to place the cutpoint can be challenging.

## 4 Conclusion

Our survivalContour software opens a new avenue for visualizing the relationship between a continuous predictor and survival outcomes. The Shiny app offers a user-friendly guided experience for many popular survival models. The full potential of our plotting approach is revealed when incorporated with state-of-the-art machine learning models through the survivalContour R package.

Importantly, the survival contour plot focuses on displaying predicted survival and does not provide insight regarding model fit. We recommend that users compute the C-index or the integrated Brier score to evaluate model fit before sharing results. Complex deep neural network-based models, like DeepHit or DeepSurv, often require significant manual tuning and the best practices for fitting such complex models are beyond the scope of this paper. Additionally, flexible models may be susceptible to overfitting, so it is always advisable to use an independent dataset for validation.

## Supplementary Material

vbae105_Supplementary_Data
