# Supplementary material for: survivalContour: visualizing predicted survival via colored contour plots
Source: Bioinform Adv. 2024 Jul 25;4(1):vbae105. doi: 10.1093/bioadv/vbae105 (PMC11290613; doi:10.1093/bioadv/vbae105)
Supplement: vbae105_Supplementary_Data [file vbae105_supplementary_data.pdf]

Supplement to "survivalContour: Visualizing predicted survival via colored contour plots"

Yushu Shi, Liangliang Zhang, Kim-Anh Do, Robert Jenq and Christine B. Peterson

1 Supported models

Model	Package
Cox model and stratified Cox model [2, 24]	survival [23]
Cox model and stratified Cox model for interval-censored data	mets [21, 8]
Parametric and spline models [20]	flexsurv [11]
Fine-Gray model for competing risks data [4]	riskRegression [6, 7]
Fine-Gray model with interval censoring [1]	intccr [19]
random survival forests [10]	randomForestSRC [9]
Cox-Time [17]<br>DeepSurv [14]<br>DeepHit [18]<br>Nnet-Survival [5]<br>PC-Hazard [16]	survivalmodels [22]

Table S1: Models and packages supported by survivalContour

2 Stratified Cox model

To demonstrate the capabilities of the survivalContour Shiny app in tandem with the stratified Cox model, we provide an illustrative example using the Veterans' Administration Lung Cancer study [12], available in the `survival` R package. The data set includes covariate data and survival outcomes for 137 subjects. Our focus is on elucidating the influence of the Karnofsky performance score [13] on survival times. Since this relationship varies by cancer subtype, we fit a stratified Cox model, with strata defined by the four subtypes in the dataset: adenocarcinoma, large cell carcinoma, small cell lung cancer, and squamous cell carcinoma. We adjusted for the additional covariates age, prior therapy status, treatment arm, and months from diagnosis to randomization. The predicted survival probabilities are shown in Figure S1, where the projected survival for each cancer type is presented in a subpanel. Higher Karnofsky scores are associated with improved survival predictions across all four subtypes: however, the strength of this association and overall hazard rates vary by cancer subtype.

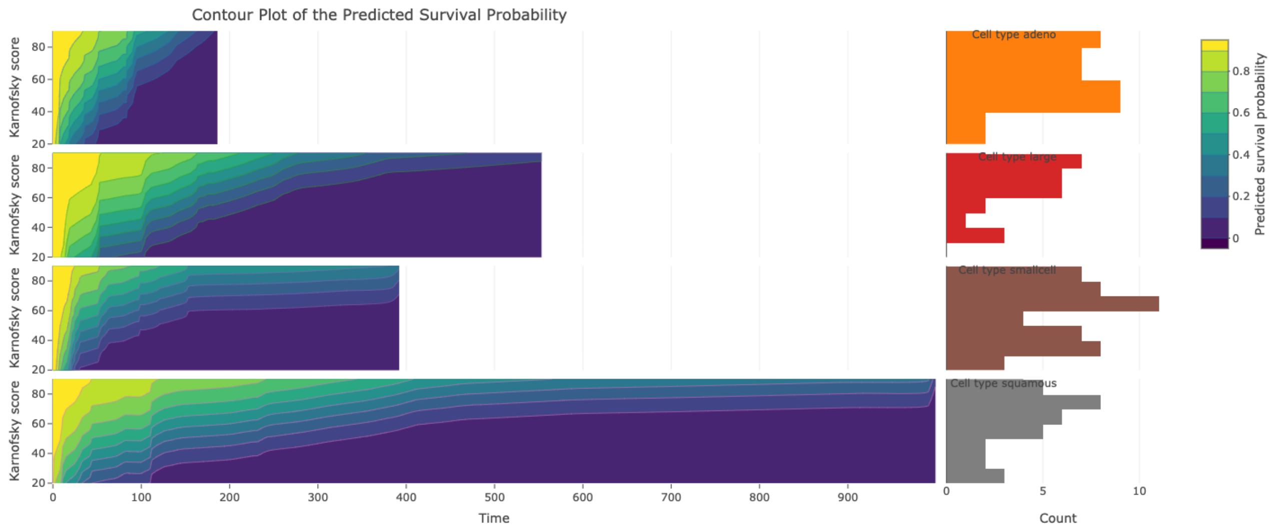

Figure S1: Plot generated by the survivalContour R package for stratified analysis. Each subpanel corresponds to a lung cancer subtype: adenocarcinoma (orange), large cell carcinoma (red), small cell carcinoma (brown), and squamous cell carcinoma (gray).

3 Competing risks

The survivalContour Shiny app, along with its accompanying R package, can handle data with competing risks. To demonstrate this capacity, we use the Paquid dataset, which was collected as part of a study on brain aging [3] and is included in the `riskRegression` package [7]. The data set includes a total of 2561 participants, with observed event times for the onset of dementia for 449 subjects and for death without dementia for 634 subjects. We focused on predicting the onset of dementia while considering death without dementia as a competing risk.

As our primary predictor variable of interest, we considered the Digit Symbol Substitution Score Test (DSST), which explores attention and psychomotor speed. Concurrently, we adjust for the Mini-Mental State Examination score (MMSE), which is a measure of overall cognitive performance. Employing the Fine and Gray model, we illustrate the influence of DSST in Figure S2 via both 2D and 3D contour representations. Here, lower scores on the DSST are associated with an increased probability of developing dementia over time, accounting for the fact that subjects that died without dementia were removed from the risk pool.

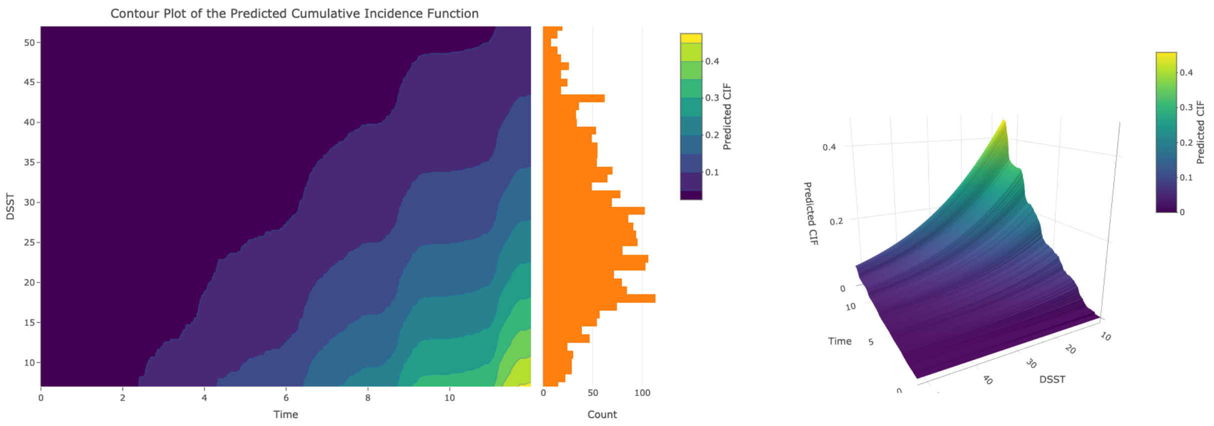

Figure S2: 2D plot (left) and 3D plot (right) generated by the survivalContour R package for the Paquid competing risks data.

4 Random survival forests

In this section, we focus on the data from the SUPPORT III study [15], which was analyzed using DeepSurv in the main text. This data set highlights a setting with a complex relationship between the continuous predictor and survival. Figure S3 depicts 2D and 3D survival contour plots showing the relationship between respiratory rate and predicted survival from a random survival forests model with 500 trees.

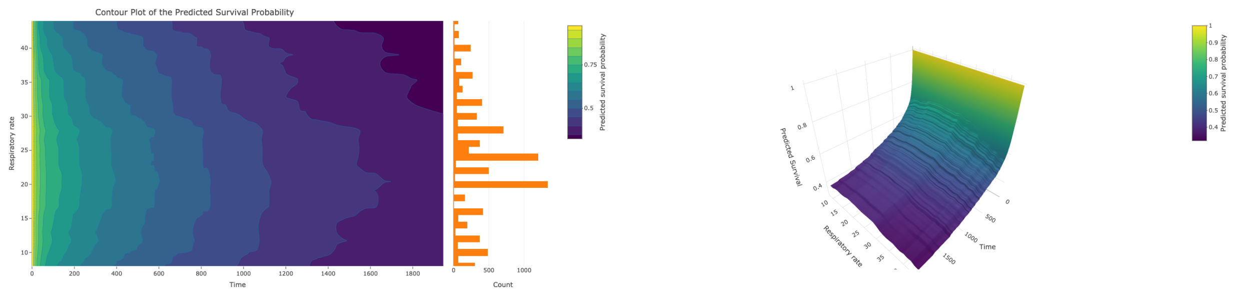

Figure S3: 2D plot (left) and 3D plot (right) generated by the survivalContour R package for SUPPORT III study using random survival forests model.

Since the random survival forest and DeepSurv utilize different approaches for prediction, the resulting contour plots are not exactly the same. Nevertheless, the non-monotone relation between respiratory rate and survival is captured by both models: survival rates deteriorate at extreme respiratory rates, whereas the median respiratory rate forecasts better survival, aligning with clinical intuition.

5 Additional figures

In addition to the colored contour plot shown in Figure 4 of the main manuscript, the Shiny app automatically generates predicted survival curves for five quantiles of the predictor values (Figure

S5). These plots provide a snapshot of the predicted survival at evenly spaced quantile values and include confidence intervals to highlight the degree of statistical uncertainty in the predictions.

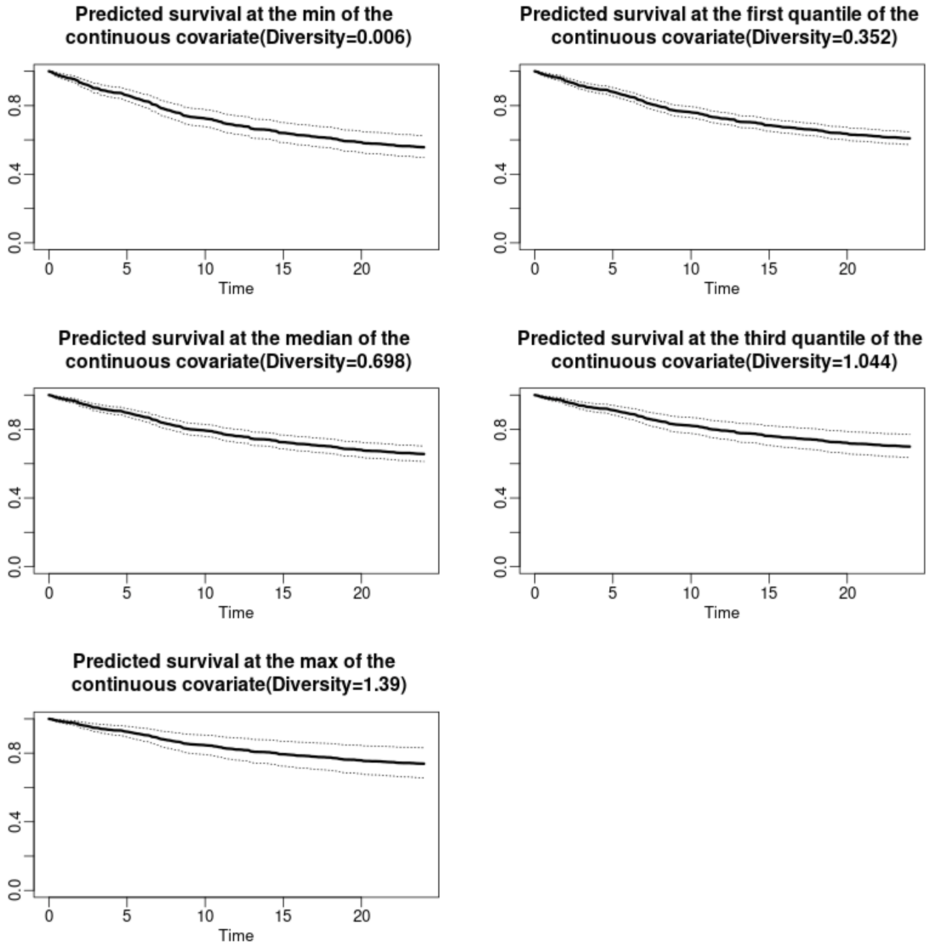

Figure S4: Predicted survival curves created using the survivalContour Shiny app. Diversity corresponds to baseline microbiome diversity on the log10 scale.

References

[1] G Bakoyannis, M Yu, and CT Yiannoutsos. Semiparametric regression on cumulative incidence function with interval-censored competing risks data. *Statistics in Medicine*, 36(23):3683–3707, 2017.

[2] DR Cox. Regression models and life-tables. *Journal of the Royal Statistical Society: Series B (Methodological)*, 34(2):187–202, 1972.

[3] JF Dartigues, M Gagnon, P Barberger-Gateau, L Letenneur, D Commenges, C Sauvel, P Michel, and R Salamon. The Paquid epidemiological program on brain ageing. *Neuroepidemiology*, 11(Suppl. 1):14–18, 04 1992.

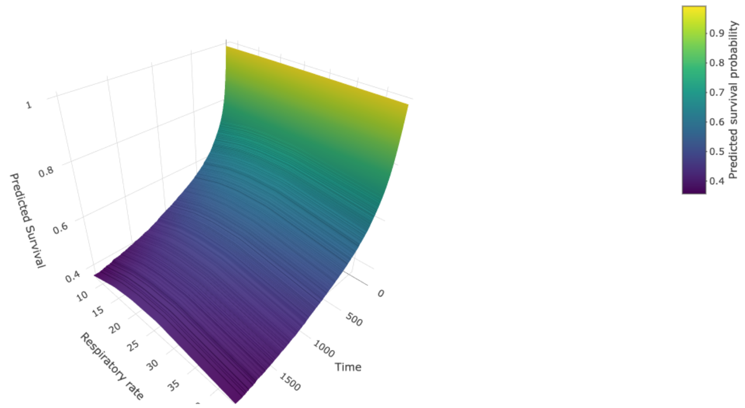

Figure S5: 3D plot generated by survivalContour to depict predictions from a DeepSurv model.

[4] JP Fine and RJ Gray. A proportional hazards model for the subdistribution of a competing risk. *Journal of the American Statistical Association*, 94(446):496–509, 1999.

[5] MF Gensheimer and B Narasimhan. A scalable discrete-time survival model for neural networks. *PeerJ*, 7:e6257, January 2019.

[6] TA Gerds and MW Kattan. *Medical Risk Prediction Models: With Ties to Machine Learning (1st ed.)*. Chapman and Hall/CRC, 2021.

[7] TA Gerds, JS Ohlendorff, and B Ozenne. *riskRegression: Risk Regression Models and Prediction Scores for Survival Analysis with Competing Risks*, 2023. R package version 2023.03.22.

[8] KK Holst, TH Scheike, and JB Hjelmborg. The liability threshold model for censored twin data. *Computational Statistics and Data Analysis*, 93:324–335, 2016.

[9] H Ishwaran and UB Kogalur. *Fast Unified Random Forests for Survival, Regression, and Classification (RF-SRC)*, 2023. R package version 3.2.2.

[10] H Ishwaran, UB Kogalur, EH Blackstone, and MS Lauer. Random survival forests. *Annals of Applied Statistics*, 2(3):841–860, 2008.

[11] C Jackson. flexsurv: A platform for parametric survival modeling in R. *J Stat Softw*, 70(8):1–33, 2016.

[12] JD Kalbfleisch and RL Prentice. *The statistical analysis of failure time data.* John Wiley & Sons, 2011.

[13] DA Karnofsky. The clinical evaluation of chemotherapeutic agents in cancer. *Evaluation of Chemotherapeutic Agents*, pages 191–205, 1949.

[14] JL Katzman, U Shaham, A Cloninger, et al. DeepSurv: personalized treatment recommender system using a Cox proportional hazards deep neural network. *BMC Med Res Methodol*, 18(1):24, 2018.

[15] WA Knaus, FE Harrell, J Lynn, et al. The SUPPORT prognostic model: Objective estimates of survival for seriously ill hospitalized adults. *Ann Intern Med*, 122(3):191–203, 1995.

[16] H Kvamme and O Borgan. Continuous and discrete-time survival prediction with neural networks. *Lifetime Data Analysis*, 27(4):710–736, 2021.

[17] H Kvamme, O Borgan, and I Scheel. Time-to-event prediction with neural networks and cox regression. *Journal of Machine Learning Research*, 20:129:1–129:30, 2019.

[18] C Lee, W Zame, J Yoon, and M van der Schaar. DeepHit: A deep learning approach to survival analysis with competing risks. *AAAI Conf Artif Int*, 32(1), 2018.

[19] J Park, G Bakoyannis, and C Yiannoutsos. Semiparametric competing risks regression under interval censoring using the r package intccr. *Computer Methods and Programs in Biomedicine*, 173:167–176, 2019.

[20] P Royston and M Parmar. Flexible parametric proportional-hazards and proportional-odds models for censored survival data, with application to prognostic modelling and estimation of treatment effects. *Stat Med*, 21(15):2175–2197, 2002.

[21] TH Scheike, KK Holst, and JB Hjelmborg. Estimating heritability for cause specific mortality based on twin studies. *Lifetime Data Analysis*, 20(2):210–233, 2014.

[22] R Sonabend. *survivalmodels: Models for Survival Analysis*, 2022. R package version 0.1.13.

[23] TM Therneau. *A Package for Survival Analysis in R*, 2023. R package version 3.5-5.

[24] TM Therneau and PM Grambsch. *Modeling Survival Data: Extending the Cox Model*. Springer, New York, 2000.
